# Excess Alcohol Use and Death among Tuberculosis Patients in the United States, 1997-2012

**DOI:** 10.4236/jtr.2016.41003

**Published:** 2016-03

**Authors:** Tyson Volkmann, Patrick K. Moonan, Roque Miramontes, John E. Oeltmann

**Affiliations:** 1 Epidemic Intelligence Service, Assigned to Division of Tuberculosis Elimination, Centers for Disease Control and Prevention, Atlanta, USA; 2 United States Public Health Service Commissioned Corps; 3 Division of Tuberculosis Elimination, Centers for Disease Control and Prevention, Atlanta, USA

**Keywords:** Substance Use, Mycobacterium, Mortality, Attributable Risk

## Abstract

**Rationale:**

Excess alcohol use (EAU) is associated with adverse TB treatment outcomes.

**Objective:**

We investigated the relationship between EAU and death among TB patients 15 years and older prescribed anti-TB treatment in the United States.

**Design:**

Using data reported to the National Tuberculosis Surveillance System for 1997-2012, we calculated adjusted odds ratios and excess attributable risk percent for death among TB patients with reported EAU.

**Results:**

EAU was associated with death among patients younger than 65. The excess attributable risk percent for death among those with reported EAU for those younger than 65 was >35%.

**Conclusions:**

Interventions to reduce EAU in patients <65 years may reduce deaths.

## 1. Introduction

In 2012, about 1.3 million people died of tuberculosis (TB) worldwide [1]. Excess alcohol use (EAU) is strongly associated with TB and may account for more than half of TB deaths in countries with a high burden of EAU, as the use of alcohol can lead to TB treatment failure and liver damage [2]. Excess alcohol use accounted for an estimated 88,000 deaths in the United States each year during 2006-2010 [3]. The association between alcohol and death during treatment for TB may be modified and confounded by a variety of factors, such as HIV and smoking [4]. Despite available national data, the stratified and adjusted associations between EAU and death during TB treatment have not been reported for the United States. The alcohol-attributable fraction for death among the entire adult population of North America has been estimated to be 14.2% for men and 3.4% for women [5]. An analysis of the excess fraction of death among TB patients with reported EAU could provide information on subgroups who could benefit the most from screening for EAU, potentially averting years of life lost. We investigated the relationship between EAU and death among TB patients aged 15 years and older prescribed anti-TB treatment in the United States.

## 2. Study Population and Methods

### 2.1. Data Collection

We used the National Tuberculosis Surveillance System (NTSS) dataset, which contains information for all cases of TB reported in the United States [6]. NTSS defines EAU as self-reported excess alcohol use within the past 12 months [7]. We included incident cases of TB disease in persons aged 15 years and older reported during 1997-2012. Data for NTSS are collected as part of routine public health practice and not for the purposes of human subjects’ research; therefore, when the study proposal was reviewed in the National Center for HIV/AIDS, Viral Hepatitis, STD, and TB Prevention, a determination was made that institutional review board approval was not required.

### 2.2. Statistical Analysis

Analysis was limited to patients with a documented response of either “yes” or “no” for EAU status and whose reason for ending anti-TB therapy was that therapy was “completed” or the patient had died during treatment. We excluded patients who never started therapy (including those who were dead at TB diagnosis), were lost to follow up, or ended therapy prematurely because of an adverse event. Since some patients with HIV infection might not be documented as HIV-infected, those who were known to be HIV-infected were considered “known positive,” whereas patients with negative or unknown status were considered as “other.”

### 2.3. Multivariate Associations between EAU and TB Outcomes

We used multivariate logistic regression analysis to assess the association of EAU and death, controlling for HIV status, race/ethnicity, and US-born origin versus foreign-born origin, covariates that could potentially modify or confound this relationship. These analyses were stratified if the associations differed significantly across strata and adjusted for confounding if the crude and adjusted estimates differed by 10% or more. Stratified adjusted odds ratios (aORs) and 95% confidence interval are displayed.

### 2.4. Excess Attributable Risk for Death Analysis

We calculated the excess attributable risk [8] percent for death among patients with reported EAU as the reason for stopping therapy for patients with TB reported in the United States. We reported the excess attributable risk percentages for death among those with reported EAU and 95% confidence intervals, stratified by gender and age. Age categories were collapsed to create a summary attributable risk for all patients under 65 years of age because those younger than 65 all shared similar attributable risks. Therefore, further stratification was not justified.

## 3. Results

Of 330,072 total cases of TB reported during the study years, 237,129 cases were included in the analysis; 26,084 (7.9%) were excluded for having missing EAU status. EAU was documented as “yes” for 36,281 (15.3%) and 21,579 (9.1%) died during antituberculosis treatment.

### 3.1. Multivariate Associations between EAU and TB Outcomes

After stratification and adjustment for confounding, EAU was significantly associated with death during treatment across some of the age, gender, and US versus foreign-born strata (**Table 1**). For males, this association was significant for the following subgroups: foreign-born men aged 15 - 64 and US-born men aged 15 - 64. For US-born men aged 65+, EAU was marginally protective. For women, the association was significant for US-born women aged 15 - 64 and foreign-born women aged 15 - 64.

### 3.2. Alcohol-Attributable Risk for TB Death

The overall excess attributable risk percent for death among TB patients with reported EAU was 10.8% (**Table 2**). Among all reported TB cases aged 15 years or older, more than 10% of the deaths that occurred during TB treatment in the United States were associated with EAU. Within the gender-specific strata, the excess attributable risk percent for death for men younger than 65 years was 35.7% (95% CI: 32.0 - 39.4), while for women younger than 65, excess attributable risk percent was 60.8% (95% CI: 54.7 - 66.9). For males and females aged 65+, the excess attributable risk percent was small and not statistically significant.

## 4. Discussion

We found that EAU was independently associated with death during TB treatment among certain subgroups in the United States, especially among foreign-born males and females. We calculated that nearly 36% of death among male TB patients younger than 65 and greater than 60% of death among female TB patients younger than 65 might be attributable to EAU. These findings concerning the excess burden of death attributable to EAU replicate results from other countries [2]. For example, EAU is attributable for 36.8% of male TB deaths in Nigeria [9], indicating that the public health threat of TB death due to EAU is a problem in disparate settings in terms of TB burden and country income level. Indeed, it is estimated that harmful alcohol use is attributable for 13% of the population attributable fraction in the 22 highest burden TB countries [10]. Age-specific estimates for the alcohol-attributable fraction for TB death are not available from any reported data, but if the global data mirror the US data, a large proportion of TB death is occurring due to EAU globally, constituting an arena for potential intervention.

This analysis was limited by some assumptions. Excess attributable risk percent calculations assume that associations are similar across subgroups. We assessed excess attributable risk percent among various age groups (e.g., age 15 - 24 and 25 - 44) and found them to be similar enough to collapse these categories. Also in the excess attributable risk percent calculations, confounding by extraneous variables was assumed not to be present. We were unable to quantify or test the unknown amount of residual confounding by smoking and other factors, but a conservative method to account for this has been to halve the effects [11]. The nature of our variable to measure alcohol use prevented the assessment of the longitudinal or cumulative effect of alcohol use. Finally, causation could not be determined due to the cross-sectional nature of the data.

A relatively large proportion of death during TB treatment was associated with EAU, especially among those younger than 65 years, indicating that the removal of EAU could avert the deaths of nearly two out of three younger women TB patients and 1 out of three among younger male TB patients. Among those older than 65, EAU was not associated with death. Those with severe alcohol use disorders may be more likely to die before the age of 65. US TB controllers should consider preferentially screening for EAU in younger age groups in order to potentially have a greater impact in the reduction of TB death among patients.

## 5. Conclusion

Interventions to reduce EAU in TB patients who are younger than 65 years of age may reduce deaths that would be attributable to EAU. These deaths in relatively young TB patients account for many years of potential life lost and the prevention of them represents an opportunity to avert a failure of the substance use and TB treatment systems in the United States.

## Figures and Tables

**Table 1:** Stratum-specific adjusted odds ratios (AORs) and 95% confidence intervals (CI) for the association between excess alcohol use and death versus completion of therapy as the reason for stopping anti-TB treatment, all, US-born, and foreign-born TB patients, United States, 1993-2012.

	All strata (n = 237,129)^a^					
	n (%)	AOR	95% CI	n (%)	AOR	95% CI
	Male (n = 147,213)			Female (n = 89,916)		
Age strata						
15 - 64	115,389 (48.7)	1.33	1.26 - 1.40	68,158 (28.7)	1.30	1.16 - 1.46
65+	31,824 (13.4)	0.92	0.84 - 1.01	21,758 (9.2)	1.01	0.76 - 1.34
US-born strata (n = 116,623)^b^						
15 - 64	57,601 (49.4)	1.16	1.09 - 1.23	26,805 (23.0)	1.25	1.11 - 1.40
65+	19,324 (16.6)	0.88	0.79 - 0.98	12,893 (11.1)	1.03	0.76 - 1.39
Foreign-born strata (n = 120,506)^b^						
15 - 64	57,788 (48.0)	1.92	1.73 - 2.15	41,353 (34.3)	2.76	1.92 - 3.97
65+	12,500 (10.4)	1.10	0.90 - 1.35	8865 (7.4)	0.84	0.36 - 1.98

a Adjusted for HIV, race/ethnicity, and US-born origin versus foreign-born origin

b Adjusted for HIV and race/ethnicity.

**Table 2:** Stratum-specific excess attributable risk percent^a^ and 95% confidence intervals (CI) for TB death, patients whose reason for stopping anti-TB therapy was either death or completing treatment, United States, 1993-2012 (n = 237,129).

	Deaths among excess alcohol users (% of all deaths in stratum)	Excess attributable percent	95% CI	Deaths among excess alcohol users (% of all deaths in stratum)	Excess attributable percent	95% CI
	Male			Female		
Age strata						
15 - 64	2544 (34.1)	35.7	32.0 - 39.4	465 (17.4)	60.8	54.7 - 66.9
65+	635 (8.8)	–0.42	–7.6 - 6.8	64 (1.5)	12.8	–7.9 - 33.5
US-born strata						
15 - 64	2055 (38.3)	3.6	–1.59 - 8.70	429 (23.7)	31.1	22.4 - 39.7
65+	506 (10.4)	–8.3	–16.6 - 0.0	58 (2.0)	6.7	–15.3 - 28.8
Foreign-born strata						
15 - 64	489 (23.3)	52.9	46.1 - 59.7	36 (4.2)	72.8	55.6 - 90.0
65+	127 (5.5)	4.4	–11.4 - 20.2	6 (0.47)	–17.8	–98.4 - 62.7

a Overall percent: 10.8%.
